# Reliability and reproducibility of the Kinvent K-push dynamometer for assessing quadriceps strength and force development in athletes and untrained individuals

**DOI:** 10.3389/fphys.2025.1573748

**Published:** 2025-06-24

**Authors:** Robert Trybulski, Jakub Więckowski, Jarosław Muracki, Filip Matuszczyk, Kamil Gałęziok, Michał Wilk, Adrian Kużdżał

**Affiliations:** ^1^ Medical Department, Wojciech Korfanty Upper Silesian Academy, Katowice, Poland; ^2^ Provita Medical Center, Żory, Poland; ^3^ National Institute of Telecommunications, Warsaw, Poland; ^4^ Department of Physical Culture and Health, Institute of Physical Culture Sciences, University of Szczecin, Szczecin, Poland; ^5^ Jerzy Kukuczka Academy of Physical Education, Katowice, Poland; ^6^ Institute of Physiotherapy Faculty of Health Sciences and Psychology Collegium Medicum, University of Rzeszów, Poland

**Keywords:** maximum voluntary contraction, rate of force development, MMA, footballer, assessment stability

## Abstract

**Objectives:**

This study aimed to (1) analyze the reliability of the Kinvent K-push handheld dynamometer for assessing quadriceps maximum voluntary contraction (MVC) and rate of force development (RFD), and (2) evaluate inter-rater reliability by assessing the reproducibility of measurements between examiners.

**Methods:**

A blinded, randomized, comparative study evaluated the reliability of quadriceps femoris MVC and RFD measurements obtained by two independent researchers. Forty-four male volunteers participated, divided into three groups based on motor skill level: elite mixed martial arts (MMA) athletes (n = 15), amateur football players (n = 15), and untrained healthy volunteers (n = 14). Three measurements per leg were taken during the experimental session, with the Kinvent K-push handheld dynamometer automatically calculating the average MVC and RFD.

**Results:**

Pearson correlations revealed that MVC and RFD measurements were highly consistent, with the left quadriceps showing nearly perfect correlations (r = 0.96 for MVC, r = 0.97 for RFD), while the right quadriceps displayed more variability, particularly in MVC. Inter-examiner comparisons showed high reproducibility, with minimal differences between the two measurers (p > 0.05). The ICC further supported this, with very high intraclass correlation values, especially for RFD (ICC = 0.999), confirming almost perfect agreement between the measurers. Group comparisons revealed that athletes, particularly MMA fighters and footballers, had significantly higher muscle strength and RFD compared to the general non-training group (p < 0.05), with athletes showing similar values for both MVC and RFD, while the general non-training group exhibited greater variability in both parameters. Bland-Altman analysis revealed strong agreement between measurers across all quadriceps measurements, with minimal systematic bias and acceptable variability, particularly in MVC assessments.

**Conclusion:**

MVC and RFD measurements in the quadriceps using the K-push handheld dynamometer are highly reliable and consistent, with minimal inter-examiner variability. These findings, in conjunction with high ICC and low MAE/MSE values, underscore the reliability of the measurement protocol used in this study. The tested instrument provides consistent and accurate results, ensuring reliable measurements across different examiners.

## 1 Introduction

Analyzing quadriceps maximum voluntary contraction (MVC) and rate of force development (RFD) is an important factor for both athletic and non-athletic populations due to its implications for health and performance (Mehmet, 2021). In athletes, understanding these parameters allows for tailored training programs to optimize power output, and speed (Behm et al., 2024). For example, sports requiring explosive movements like sprinting or jumping heavily rely on high RFD (Buckthorpe, 2017). In non-athletic individuals, assessing quadriceps MVC and RFD can help identify age-related muscle decline (sarcopenia), neurological disorders, or injuries affecting muscle function (Schimidt et al., 2014). This information aids in developing targeted interventions to improve mobility, balance, and independence in daily living. Moreover, optimizing quadriceps strength and RFD is essential for knee joint stability, reducing the risk of osteoarthritis and falls in older adults (Alshahrani and Reddy, 2023). The measurements which can be made by the HHDs such as Kinvent K-Push can be helpful in injury prevention by measuring and assessing the force ratio between flexors and extensors which is proven to be crucial for the injury risk assessment especially for the knee joint (Esmaeili and Sharifi, 2022; Huang et al., 2024; Śliwowski et al., 2024).

To measure quadriceps MVC, participants are typically asked to perform a maximal isometric knee extension against a fixed resistance, with the force produced recorded by a force transducer or dynamometer (Berger et al., 2012). The highest force value achieved during several attempts is considered the MVC (Morton et al., 2005). For RFD, the same setup is used, but the focus is on the initial slope of the force-time curve, reflecting the speed at which force increases from the onset of contraction (Maffiuletti et al., 2016). The gold standard for measuring MVC and RFD is considered to be the use of an isokinetic dynamometer, which allows for controlled and precise measurement of force and angular velocity throughout the range of motion (Jørgensen et al., 2017). Alternative approaches include handheld dynamometers, which are more portable and affordable but may have limitations in accuracy and reliability (Takeda et al., 2018).

Handheld dynamometers (HHD) offer a practical and affordable way to assess muscle strength, particularly in clinical settings. Their portability allows for easy testing in various locations, and they can provide objective measurements compared to manual muscle testing (Conable and Rosner, 2011). However, HHDs have limitations. They may not be as accurate as isokinetic dynamometers, especially for assessing larger muscle groups or dynamic movements (Kolber and Cleland, 2005). The reproducibility of HHD measurements can also be influenced by the examiner’s experience and technique (Schrama et al., 2014). Additionally, the force exerted by the examiner to stabilize the device can inadvertently contribute to the measured force, potentially overestimating the patient’s actual strength (Bohannon, 2019). This is particularly relevant when the patient’s strength exceeds the examiner’s ability to provide counter-resistance. Despite these limitations, HHDs remain a valuable tool for muscle strength assessment when used appropriately and interpreted cautiously (Kittelson et al., 2021).

Assessing the reliability of HHDs and the reproducibility of examiners is crucial for ensuring the accuracy and consistency of muscle strength measurements in clinical practice. Reliability refers to the consistency of measurements obtained by the same examiner or device over time (Hopkins et al., 2001), while reproducibility assesses the agreement between measurements taken by different examiners or devices (McAlinden et al., 2015). If an HHD demonstrates poor reliability or if examiners exhibit low reproducibility, it can lead to inconsistent and unreliable data, compromising the validity of clinical assessments and potentially affecting treatment decisions.

By establishing the reliability and reproducibility of HHDs, clinicians can have confidence in the accuracy of their measurements and make informed decisions regarding patient care. This is particularly important when tracking changes in muscle strength over time, such as during rehabilitation programs or when monitoring disease progression (Lee et al., 2017). In our study focusing on the Kinvent force dynamometer, a new product in the market, establishing its reliability and examiner reproducibility is essential for determining its value in clinical practice. If the Kinvent HHD demonstrates strong reliability and reproducibility, it can be considered a valuable tool for clinicians seeking an objective and consistent method for assessing muscle strength. This information can help clinicians confidently integrate the Kinvent HHD into their practice, ultimately leading to improved patient care.

This study aimed to determine the reliability and validity of the Kinvent Force dynamometer for measuring MVC and RFD in the quadriceps femoris of individuals with varying motor skill levels.

## 2 Materials and methods

### 2.1 Study design

A comparative, experimental study was conducted using two blinded researchers to assess the maximum voluntary contraction (MVC) and rate of force development (RFD) of the right and left quadriceps femoris muscles. The order of thigh measurements (right or left first) was randomized (1:1 ratio) for each participant using randomizer.org to ensure unbiased allocation. One researcher performed the initial assessment, followed by a 5-min rest period, after which the second researcher conducted the subsequent assessment. Neither researcher had access to the data recorded by a third, independent researcher (a student). Participants were instructed to refrain from training or strenuous activity for 24 h prior to the assessment. The testing environment maintained consistent temperature and humidity levels. All testing took place between 9:00 a.m. and 12:00 p.m. Ethical approval was granted by the National Council of Physiotherapists (Ref. No. 3.03.2024), and the study was registered with the ISRCTN registry (https://doi.org/10.1186/ISRCTN15418049). The study adhered to the principles outlined in the Declaration of Helsinki.

### 2.2 Participants

Participants were required to have a dominant right leg, be between 18 and 45 years old, and have at least 5 years of experience in their respective sport, training at least three times per week (with the exception of the untrained group). The dominant lower limb was determined by the declaration of the participants. Inclusion criteria also required participants to have no history of any severe injuries, be free of any musculoskeletal injuries within 3 months prior to the study, have no history of surgical procedures on the knee, thigh, or hip, and be in good general health. McKay’s participant classification scheme was used to categorize participants at levels 2, 3, and 4, corresponding to highly trained/national level athletes (McKay et al., 2022). Individuals were excluded if they had high blood pressure (>140/90 mmHg) before testing, current injuries, or were using pain medication or other substances that could alter muscle tone. Exclusion criteria also included the history of severe injuries and current presence of extreme fatigue, fever, infection, or withdrawal from the study at the participant’s request. Feeling pain during maximal contraction of the quadriceps was another exclusion criteria which would eliminate a participant if occurred during testing. Written informed consent was obtained from all participants before their inclusion in the study, along with a health questionnaire. Participants were free to withdraw from the study at any time. The authors affirm that they have obtained written consent from the participants to use their image in this scientific article.

Forty-four male volunteers participated in this study, divided into three groups based on motor skill level and sport (Table 1): elite mixed martial arts (MMA) athletes (n = 15), amateur football players (n = 15), and untrained healthy volunteers (n = 14). To determine the necessary sample size, we conducted a power analysis. We estimated an effect size of 0.932. With an alpha of 0.05 and a desired power of 0.95, the analysis indicated a sample size of six participants.

**TABLE 1:** Comparative analysis of physical characteristics across three examined groups presenting number of samples (n), mean value within each group (Mean), and standard deviation (SD).

Group	Age (years)		Body mass (kg)		Height (cm)		BMI (kg/m^2^)		Training experience (years)	
	Mean	SD	Mean	SD	Mean	SD	Mean	SD	Mean	SD
General non-training group (n = 14)	32.50	7.32	75.92	6.81	179.4	6.30	23.45	1.45	0.00	0.00
Footballers (n = 15)	29.60	7.61	79.26	7.66	179.1	4.60	24.71	1.50	11.93	4.61
MMA fighters (n = 15)	28.06	8.22	83.73	7.60	180.8	4.95	25.63	2.26	9.00	3.82

BMI: body mass index; MMA: mixed martial arts; SD: standard deviation.

Demographic and physical characteristics were compared across groups, including age, training experience, weight, height, and body mass index (BMI). MMA fighters had the youngest average age (28.06 years), followed by football players (29.60 years) and the general population (32.50 years). MMA fighters also reported the least training experience (9 years), while football players averaged 11.93 years. As expected, the general population reported no professional training experience. MMA fighters had the highest mean weight (83.73 kg), followed by football players (79.26 kg) and the general population (75.92 kg). Height differences were minimal: MMA fighters averaged 180.8 cm, the general population 179.4 cm, and football players 179.1 cm. MMA fighters had the highest mean BMI (25.63 kg/m^2^), followed by football players (24.71 kg/m^2^) and the general population (23.45 kg/m^2^). These findings highlight the diverse physical profiles associated with different sports and demonstrate how athletic demands influence demographic and physiological characteristics. Table 1 presents a detailed comparison of physical characteristics across groups.

### 2.3 Instrument

The Kinvent K-Push Dynamometer (370 g, 90 kgF maximum force capacity, 0.1% reading precision; 1000 Hz sampling rate, Kinvent Physio, Montpellier, France) is a versatile, portable device designed for real-time force measurement and feedback in various settings, including physiotherapy, rehabilitation, sports performance, and biomechanical research. It measures forces in multiple planes (e.g., compression, tension) and provides precise data for strength assessments, such as maximum voluntary contraction (MVC), rate of force development (RFD), and functional evaluations. Utilizing Bluetooth technology, the K-Push seamlessly connects to smartphones, tablets, or computers. The Kinvent Physio app enables real-time data visualization, displaying peak force, average force, and force-time curves (Olds et al., 2023). The device calibrates automatically, and no errors occurred during measurement.

### 2.4 Measurements

**FIGURE 1:**
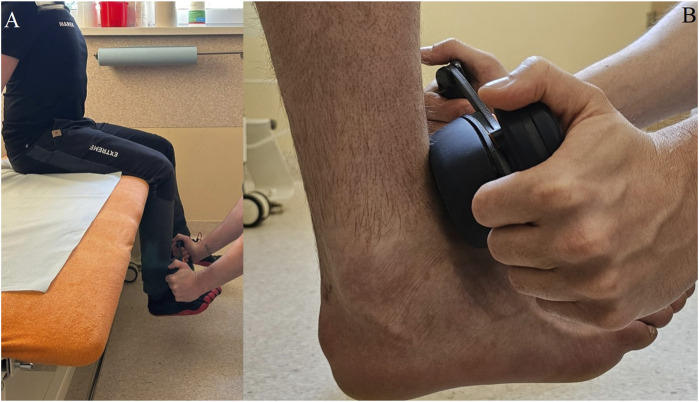
Test position of the Kinvent K-Push handheld dynamometer during testing procedure **(A)** and the measurement point **(B)**.

One day prior to the experimental trial, each participant completed a familiarization session, which included 3 repetitions of single 5-s isometric contraction of the quadriceps femoris muscles to measure muscle strength for each leg. Before both the familiarization and experimental sessions, participants received training on the correct testing position and performed a warm-up. The testing position involved participants sitting on a medical couch with their legs hanging down without touching the ground, knees and hips flexed at 90°. The torso was maintained in an upright position supported by a solid box leaning against the wall, with arms extended and holding onto the medical table for support and ensuring stable body position (Figures 1A,B). The participants were instructed to sit upright and lean against the backrest.

The warm-up consisted of two sets of 10 squats at a comfortable pace, with a 30-s rest between sets, followed by three sets of static quadriceps femoris stretches. Each stretch involved pulling the heel towards the buttock while standing and holding onto the back of a chair for support, with each stretch held for 15 s. The break between the warm-up and testing lasted 3 min. During the experimental session, measurements were taken three times for each leg, with the Kinvent Physio app (v.2.12.0, Kinvent, Montpellier, France) software automatically calculating the average. The researcher held the dynamometer in both hands with their elbows straight and braced against the wall directly in front of the subject and took the measurement, being careful not to use any force to counteract the participant’s movement or to lose position. The dynamometer was applied to the lower limb in contact with the tibia at the height of the medial malleolus, and then the comfort of the participant was checked by isometric tension to ensure that he was not inhibited by pain related to pressure on the uncomfortable spot during the test. Participants were instructed to exert maximum effort during the isometric test. The measurer verbally encouraged all participants during all trials using standard commands (push, keep pushing, stop). Before performing the test, the measurer told each participant individually the following formula:

The test will show us your maximum knee extension force. When I say “push,” you will start pushing as hard as you can until I say “stop.” Before each test I will ask “are you ready?” and then the command “push” will be given. Immediately stop pushing if you feel any pain or discomfort during the test. Do you have any questions?

The test setup included 5 s of preparation time from the moment the measurement is turned on, 5 s of maximum contraction time, 20 s of rest between the 3 repetitions for each leg. Then the subjects were switched and after a 5-min rest the patient performed the same test with the second measurer.

#### 2.4.1 Rate of force development (RFD)

RFD was calculated from the force-time data collected by the Kinvent K-Push dynamometer during the isometric quadriceps contractions. The RFD (kg/s) was determined by calculating the change in force (ΔF) over a specific 100 ms time interval (Δt) from the onset of the contraction. This involved identifying the point at which force began to increase and then calculating the slope of the force-time curve within a defined window of 0–100 ms from that point. The Kinvent software automatically processed the force-time data to derive the RFD (kg/s) values.

#### 2.4.2 Maximum voluntary contraction (MVC)

MVC was determined from the peak force value recorded during the isometric quadriceps contractions using the Kinvent K-Push dynamometer. Participants were instructed to exert their maximal effort, and the highest force value achieved during the three trials for each leg was taken as the MVC. The Kinvent software automatically captured and stored the peak force (kg) data for each trial.

### 2.5 Statistical analysis

Normality and homogeneity of the sample was preliminary inspected (p > 0.05). To evaluate the agreement between measurements taken by two specialists, we employed several statistical methods. We first assessed the linear relationship between the two sets of measurements using the Pearson correlation coefficient (r). The magnitude of the correlation was interpreted according to the following scale: 0.0–0.1, trivial; 0.1–0.3, small; 0.3–0.5, moderate; 0.5–0.7, large; 0.7–0.9, very large; and greater than 0.9, nearly perfect.

Intraclass Correlation Coefficients (ICCs) were used to assess the reliability and agreement between the two raters’ measurements of muscle characteristics. A three-model approach (ICC1,2, ICC2,2, and ICC3,2) was employed to evaluate various aspects of rater consistency, with 95% confidence intervals calculated for each ICC value to determine the stability and range of agreement.

To further quantify measurement reliability, additional statistical metrics were computed, including the Standard Error of Measurement (SEM), Coefficient of Variation (CV%), Minimal Detectable Change (MDC), and Smallest Worthwhile Change (SWC). SEM was derived from ICC2 values to assess absolute reliability, while CV% measured relative variability across trials. MDC was calculated to determine the smallest measurable change beyond random variation, and SWC represented the minimum meaningful clinical difference based on between-subject variability. Additionally, Bland-Altman plots with 95% limits of agreement were used to assess any systematic bias between the two examiners, providing a visual representation of the agreement and identifying potential biases or inconsistencies in measurements.

Beyond correlation, we used the mean absolute error (MAE) to quantify the average magnitude of the differences between paired measurements, providing a direct measure of agreement in the original units. To further examine the distribution of discrepancies and emphasize larger errors, we calculated the mean squared error (MSE). The MSE, by squaring the errors, gives greater weight to larger differences, making it sensitive to outliers. By considering these metrics together, we aimed to provide a comprehensive assessment of both the degree of linear association and the magnitude and distribution of discrepancies between the specialists’ measurements.

## 3 Results

The study involved 44 participants who underwent measurements targeting specific muscle groups. Two muscles were analyzed: the Left and Right quadriceps, with each muscle assessed in two areas: Rate of Force Development (RFD) and Maximum Voluntary Contraction (MVC). As a result, a total of 176 measurements were taken (44 * 2 = 88 by one specialist, 44 * 2 * 2 = 176 by two specialists). Participants came from three groups: the general population (n = 14), footballers (n = 15), and MMA fighters (n = 15).

**FIGURE 2:**
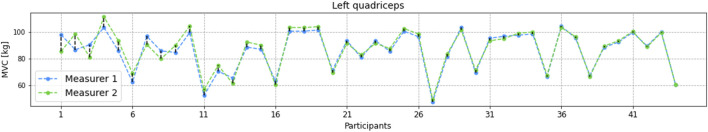
Comparative analysis of MVC measurements for the Left quadriceps across participants, performed by two measurers, sorted in descending order based on the measurement differences between measurers.

**FIGURE 3:**
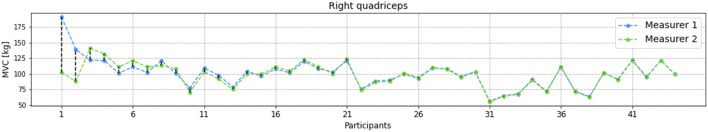
Comparative analysis of MVC measurements for the Right quadriceps across participants, performed by two measurers, sorted in descending order based on the measurement differences between measurers.

**FIGURE 4:**
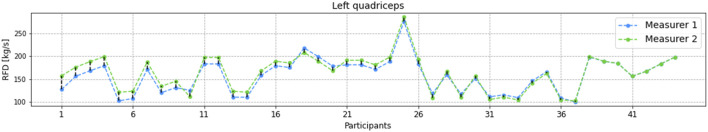
Comparative analysis of RFD measurements for the Left quadriceps across participants, performed by two measurers, sorted in descending order based on the measurement differences between measurers.

**FIGURE 5:**
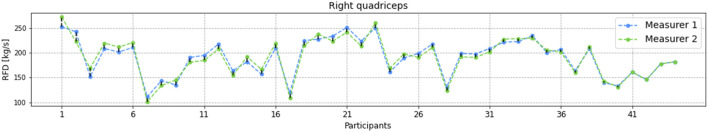
Comparative analysis of RFD measurements for the Right quadriceps across participants, performed by two measurers, sorted in descending order based on the measurement differences between measurers.

To assess the impact of different measurers on the correlation of results, the data from the various groups were combined into a single vector. This method allowed for a comprehensive analysis of the correlations, focusing particularly on the differences due to the measurer’s expertise or technique. The statistical analysis was designed to address three scenarios: individual assessments for each muscle (Left and Right quadriceps), and joint analyses incorporating measurements from both muscles. These scenarios facilitated an exploration of measurement correlations in different contexts, offering insights into the consistency and reliability of the evaluation process. These results as presented on graphs show in differences between measurer 1 and 2 for each participant in MVC and RFD of the left and right quadriceps muscles (Figures 2–5).

The results presented in Table 2provide an in-depth look at the consistency and relationship between measurements of the Left and Right quadriceps for MVC and RFD, using selected metrics.

**TABLE 2:** Comparative analysis of correlation measures [Pearson, Mean Absolute Error (MAE), Mean Absolute Error divided by the average value (MAE %), and Mean Squared Error (MSE)] between examined cases of muscles performance measurements [maximum voluntary contraction (MVC) and rate of force development (RFD)] of left quadriceps, right quadriceps and both.

Statistical parameter	MVC (kg)			RFD (kg/s)		
	Left quadriceps	Right quadriceps	Both	Left quadriceps	Right quadriceps	Both
Pearson (r)	0.9589	0.7185	0.8181	0.9651	0.9715	0.9696
MAE	3.3112	6.5477	4.9295	9.4772	8.1136	7.7954
MAE %	3.8257	6.5915	5.3037	5.9119	4.2477	5.0071
MSE	19.1675	265.0774	142.1225	132.2954	84.9773	108.6364

MAE: mean absolute error; MSE: mean squared error; MVC: maximum voluntary contraction; RFD: rate of force development.

The Pearson correlations revealed strong to very strong relationships between measurements. For MVC, the left quadriceps showed a near-ideal correlation (r = 0.9589), indicating very high consistency between measurers on the left side. The right quadriceps exhibited a very high correlation (r = 0.7185), indicating more variability in measurements on the right side. RFD correlations were robust for both sides, with the left quadriceps (r = 0.9651) and right quadriceps (r = 0.9715) showing strong consistency, and an overall r = 0.9696, reflecting near-ideal agreement.

The MAE values confirmed these patterns. For MVC, right quadriceps had a significantly higher MAE (6.5477) than the left quadriceps (3.3112), highlighting greater variability and error on the right side. For RFD, the left quadriceps had a higher MAE (9.4772) compared to the right quadriceps (8.1136), suggesting slightly greater error variability on the left side. These findings were consistent in MAE percentages, with right MVC showing 6.59% error, compared to 3.83% for left MVC, and left RFD having 5.91% error versus 4.25% for right RFD.

The MSE analysis further supported these differences. For MVC, the right quadriceps (265.0774) exhibited substantially larger MSE compared to the left quadriceps (19.1675), indicating greater measurement error on the right side. Similarly, for RFD, the left quadriceps (132.2954) had a higher MSE than the right quadriceps (84.9773), though the difference was less pronounced. These trends were also visually confirmed through comparative analysis, which highlighted greater measurement variability in right MVC and left RFD, suggesting potential areas for improving measurement consistency.

The second part of the statistical analysis examined correlations and differences between groups. Measurements for each group were analyzed separately. Including varied groups allowed assessment of muscle characteristics across athletic disciplines and demographics. Due to unequal group sizes, a systematic approach ensured fair comparisons. For each muscle characteristic (MVC or RFD), a grand mean was calculated from all measurements. Group-specific means were then calculated. The absolute difference between two group means was divided by their average to express the difference as a percentage. This method highlighted percentage differences in muscle properties across groups, adjusting for unequal sample sizes, which standard statistical methods assume. This procedure standardized differences between group averages for equitable analysis.

Table 3 higher part presents matrices comparing groups based on left quadriceps MVC and RFD. Footballers and MMA fighters showed minimal MVC difference (0.38%), indicating high similarity. The general non-training group differed substantially, with 16.66% difference from footballers and 17.03% from MMA fighters. RFD showed a similar trend. Footballers and MMA fighters had small RFD differences (3.37%), slightly higher than for MVC. The general non-training group showed larger differences, 21.07% from footballers and 24.27% from MMA fighters. Non-athletes showed more variability, particularly compared to MMA fighters. The two sports groups were highly similar for MVC and RFD, while the general non-training group showed noticeable variation.

**TABLE 3:** Comparative analysis of correlation between examined groups regarding the Left quadriceps/Right Quadriceps and MVC and RFDs parameters calculated as difference between average value of a selected parameter of the selected muscle of two groups divided by the mean of those averages. Values in matrices are symmetric so only space above diagonal was filled.

Sport group	General non-training group	Football players	MMA fighters
MVC – Left Quadriceps			
General non-training group	0.00%	16.66%	17.03%
Football players		0.00%	0.38%
MMA fighters			0.00%
RFD – Left Quadriceps			
General non-training group	0.00%	21.07%	24.27%
Football players		0.00%	3.37%
MMA fighters			0.00%
MVC – Right Quadriceps			
General non-training group	0.00%	17.48%	15.69%
Football players		0.00%	1.83%
MMA fighters			0.00%
RFD – Right Quadriceps			
General non-training group	0.00%	16.44%	22.03%
Football players		0.00%	5.79%
MMA fighters			0.00%

Table 3 lower part shows comparison of groups based on right quadriceps MVC and RFD. Trends mirrored left quadriceps results, with some variations. For MVC, footballers and MMA fighters differed by 1.83%, slightly less than for left quadriceps, again showing high similarity. The general non-training group differed more, 17.48% from footballers and 15.69% from MMA fighters, suggesting greater MVC variability in non-athletes. For RFD, footballers and MMA fighters differed by 5.79%, more than for left quadriceps, indicating some RFD variability between these athletes. The general non-training group differed more, 16.44% from footballers and 22.03% from MMA fighters, suggesting greater variability in explosive force development in non-athletes, particularly compared to MMA fighters. Right quadriceps results reinforced left quadriceps findings: the two sports groups remained highly similar for MVC and RFD, while the general non-training group showed noticeable variation, especially compared to MMA fighters, highlighting the distinct muscle characteristics required for this discipline.

Table 4 presents ICCs for MVC and RFD in left and right quadriceps across three models (ICC1,2, ICC2,2, and ICC3,2). Left quadriceps MVC reliability was very high (ICC = 0.998–0.999), with narrow confidence intervals (0.970–1.000), indicating strong consistency. Left quadriceps RFD reliability was also very high (ICC = 0.999), though with slightly wider confidence intervals (0.640–1.000) for ICC2,2, suggesting slightly more variability. Overall, left quadriceps MVC and RFD showed high agreement between measurers.

**TABLE 4:** Reliability coefficients (Intraclass Correlation Coefficients, ICC) with 95% confidence intervals (CI) for MVC and RFD measurements of the Left and Right quadriceps across three models (ICC1,2, ICC2,2, ICC3,2).

Muscle and characteristic	Reliability coefficients (95% CI)		
	ICC1,2	ICC2,2	ICC3,2
Left quadriceps			
MVC	0.999 (0.990–1.000)	0.999 (0.970–1.000)	0.998 (0.980–1.000)
RFD	0.999 (0.900–1.000)	0.999 (0.604–1.000)	0.999 (0.920–1.000)
Right quadriceps			
MVC	0.990 (0.850–1.000)	0.990 (0.830–1.000)	0.993 (0.720–1.000)
RFD	0.999 (1.000–1.000)	0.999 (1.000–1.000)	0.999 (1.000–1.000)

MVC: maximum voluntary contraction; RFD: rate of force development.

Right quadriceps MVC reliability was slightly lower but still strong (ICC = 0.990–0.993), with confidence intervals (0.720–1.000) suggesting slightly less stability. Right quadriceps RFD reliability was near-ideal (ICC = 0.999), with perfect agreement indicated by confidence intervals (1.000–1.000).

Both muscles demonstrated high reliability, with RFD showing particularly strong consistency, while MVC, especially in the right quadriceps, showed slightly more variability.

Table 5 demonstrates reliability for all quadriceps measurements, with ICC2 values exceeding 0.98, indicating strong agreement between raters. MVC measurements show lower variability (CV%: 17.46%–21.43%) compared to RFD (CV%: 20.10%–23.89%), reflecting the inherent stability of MVC and the greater fluctuation in RFD. Right Quadriceps RFD has the highest reliability (ICC = 0.9999) and lowest measurement error (SEM = 0.3603), suggesting high precision.

**TABLE 5:** Reliability metrics based on (Intraclass Correlation Coefficients, ICC) calculated for ICC2 for MVC and RFD measurements of the Left and Right quadriceps.

Muscle and characteristic	ICC2,2	SD	Mean	SEM	CV%	MDC	SWC
Left quadriceps							
MVC	0.9982	15.1122	86.5557	0.6464	17.4595	1.7917	3.1736
RFD	0.9881	38.2909	160.3068	1.1810	23.8860	11.5890	7.8600
Right quadriceps							
MVC	0.9813	21.2909	99.3352	2.9095	21.4333	8.0648	3.6131
RFD	0.9999	38.4003	191.0113	0.3603	20.1037	0.9986	8.3408

MVC: maximum voluntary contraction; RFD: rate of force development; SD: standard deviation, SEM: standard error of measurement; CV%: coefficient of variation; MDC: minimal detectable change; SWC: smallest worthwhile change.

Measurement precision, assessed via SEM and MDC, shows that Right Quadriceps RFD has the lowest MDC (0.9986), meaning that even small meaningful changes can be detected, whereas Left Quadriceps RFD has the highest MDC (11.5890), indicating greater measurement noise. Comparing MDC to SWC, Right Quadriceps RFD is highly sensitive, while Left Quadriceps RFD’s MDC exceeds its SWC, making it harder in detecting meaningful clinical changes.

MVC measures are more stable than RFD, and for the Right Quadriceps RFD was the most precise and sensitive measurement, whereas Left Quadriceps RFD may require methodological refinements to improve sensitivity.

**FIGURE 6:**
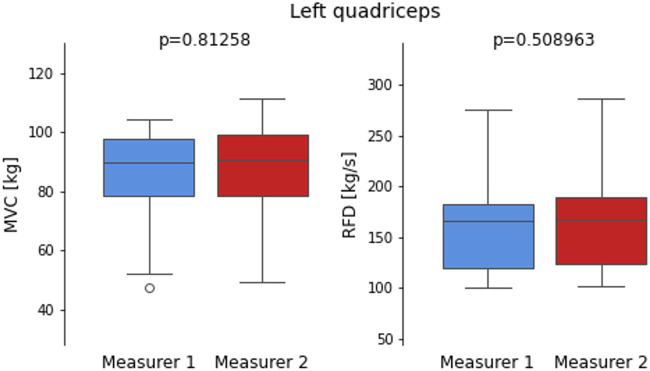
Boxplots comparing maximum voluntary contraction (MVC) and Rate of Force Development (RFD) measurements for the Left quadriceps recorded by two measurers.

**FIGURE 7:**
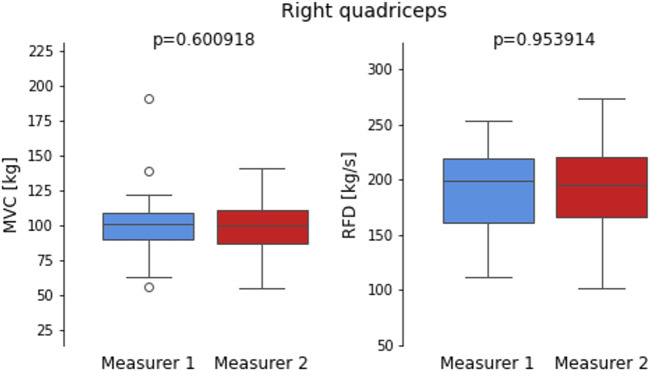
Boxplots compare maximum voluntary contraction (MVC) and Rate of Force Development (RFD) measurements for the right quadriceps recorded by two measures.

Figures 6, 7 compare the MVC and RFD measurements for both the Left and Right quadriceps, taken by two measurers. The boxplots visually depict the distribution of values for each muscle characteristic, highlighting the reliability and consistency between the two measurers. The p-values derived from independent t-tests indicate no statistically significant differences in measurements between the two measurers for both quadriceps and muscle characteristics.

For the Left quadriceps, both MVC and RFD show nearly identical distributions between Measurer 1 and Measurer 2. The p-values for MVC (p = 0.81258) and RFD (p = 0.508963) indicate no significant differences, supporting the earlier findings of high reliability (ICC >0.98) for both characteristics. These results align with the low measurement variability observed in the MAE and MSE analyses for the Left quadriceps, confirming excellent consistency between the measurers.

Similarly, for the Right quadriceps, the MVC measurements show slightly more variability compared to the Left quadriceps, but the p-value (p = 0.600918) remains non-significant, suggesting minimal differences between the two measures. The RFD measurements for the Right quadriceps display an even closer alignment, with a p-value of 0.953914, indicating almost identical results. These findings are consistent with the very high ICC values and low MAE observed for RFD, particularly on the Right side. Together, the boxplots visually reinforce the overall high reliability and consistency observed across the measures for both MVC and RFD measurements, supporting the robustness of the reliability metrics.

Table 6 compares left and right quadriceps muscle strength (MVC and RFD) across groups. Mean values were calculated from two measures per group. Absolute difference and percentage difference (relative to the overall mean) were calculated. Paired t-tests assessed statistical significance.

**TABLE 6:** Comparison of differences in muscle strength parameters (MVC and RFD) between the Left and Right quadriceps across three groups: General non-training group, Footballers, and MMA fighters.

Group and parameter	Mean - left quadriceps	Mean - right quadriceps	Absolute difference	Absolute difference (%)	Paired t-test (p-value)
General non-training group					
MVC	67.82	78.13	10.31	14.13	−5.49 (0.0001)*
RFD	114.96	143.96	29.50	22.83	−6.13 (0.0003)*
Footballers					
MVC	94.93	111.23	16.30	15.81	−3.99 (0.0013)*
RFD	175.57	200.63	25.07	13.33	−6.95 (0.0001)*
MMA fighters					
MVC	95.66	107.23	11.56	11.40	−8.83 (0.0000)*
RFD	187.83	225.30	37.47	18.14	−4.24 (0.0008)*

MVC: maximum voluntary contraction; RFD: rate of force development.

The general non-training group showed the largest relative imbalance for both MVC (10.31 kg, 14.13%, p = 0.0001) and RFD (29.50 kg/s, 22.83%, p = 0.0003), indicating significant asymmetry. Footballers had a larger absolute MVC difference (16.30 kg, 15.81%, p = 0.0013) but smaller relative difference, and a significant RFD imbalance (25.07 kg/s, 13.33%, p = 0.0001). MMA fighters showed the lowest MVC asymmetry (11.56 kg, 11.40%, p < 0.0001), suggesting high symmetry likely due to sport demands, but a significant RFD imbalance (37.47 kg/s, 18.14%, p = 0.0008).

One-way ANOVAs were conducted to explore group differences in MVC and RFD for both the left and right quadriceps. The results indicated significant differences across groups for both muscle characteristics in both limbs.

For the Left quadriceps MVC, the F-statistic was 58.82 (p < 0.001), indicating significant group differences, suggesting that the groups differed considerably in their MVC values. Similarly, for the Right quadriceps MVC, the F-statistic was 25.73 (p < 0.001), also indicating significant differences across the groups. For the Left quadriceps RFD, the F-statistic was 50.31 (p < 0.001), showing significant differences between groups, while for the Right quadriceps RFD, the F-statistic was 77.69 (p < 0.001), further confirming significant variability in RFD values across the groups.

Post-hoc Tukey’s HSD tests identified specific differences. For left quadriceps MVC, the general non-training group had significantly lower values than both footballers and MMA fighters (mean difference = −27.11 and −27.84, p < 0.001), but no significant difference existed between the athletic groups. For left quadriceps RFD, the general non-training group was again lower than both athletic groups (mean difference = −61.10 and −73.37, p < 0.001), with no significant difference between athletes.

Right quadriceps showed a similar MVC trend: the general non-training group was lower than both footballers and MMA fighters (mean difference = −33.10 and −29.09, p < 0.001), with no significant difference between athletes. For right quadriceps RFD, all comparisons were significant: the general non-training group was lower than both footballers and MMA fighters (mean difference = −56.67 and −81.34, p < 0.001), and footballers were lower than MMA fighters (mean difference = −24.67, p = 0.0015).

Athletes had significantly higher muscle strength in both quadriceps compared to the general non-training group. While left quadriceps differences between athletes were less pronounced, MMA fighters showed greater right quadriceps RFD, suggesting sport-specific adaptations in explosive power.

Next step of the analysis included Bland-Altman plots, which are a widely used method for assessing agreement between two measurement techniques or raters, providing a visual representation of systematic bias and variability. These plots compare the mean of two measurements against their difference, allowing for the identification of potential discrepancies. The key components include the mean difference, which indicates systematic bias, and the 95% limits of agreement (LoA), which define the range within which most differences between the two measurements are expected to fall. By incorporating these elements, Bland-Altman analysis offers a comprehensive evaluation of measurement reliability, complementing other statistical metrics such as ICC, MAE, and MSE.

**FIGURE 8:**
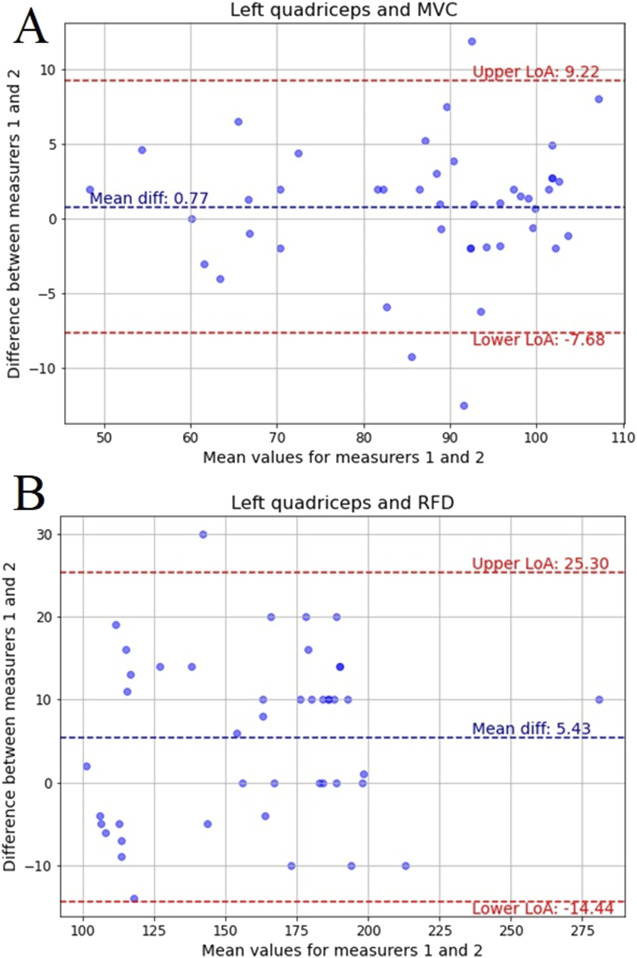
Bland-Altman plots for: **(A)** MVC of the left quadriceps, **(B)** RFD of the left quadriceps recorded by two measurers with mean difference and limits of agreement (LoA) for 95%.

Figure 8A illustrates the Bland-Altman plot for MVC measurements of the left quadriceps, showing a small mean difference of 0.77, with 95% LoA from −7.68 to 9.22. These results suggest minimal systematic bias between measurers and strong agreement. This aligns with the high reliability observed in ICC values and the nearly identical distributions seen in the boxplots (Figures 6, 7), reinforcing the consistency of MVC measurements in the left quadriceps.

Figure 8B presents the Bland-Altman plot for RFD measurements of the left quadriceps, with a mean difference of 5.43 and LoA ranging from −14.44 to 25.30. Although the agreement remains high, the wider LoA suggests slightly greater variability between measurers compared to MVC. This is consistent with the MAE and MSE results, which indicated slightly higher variability in RFD measurements, though still within an acceptable range.

**FIGURE 9:**
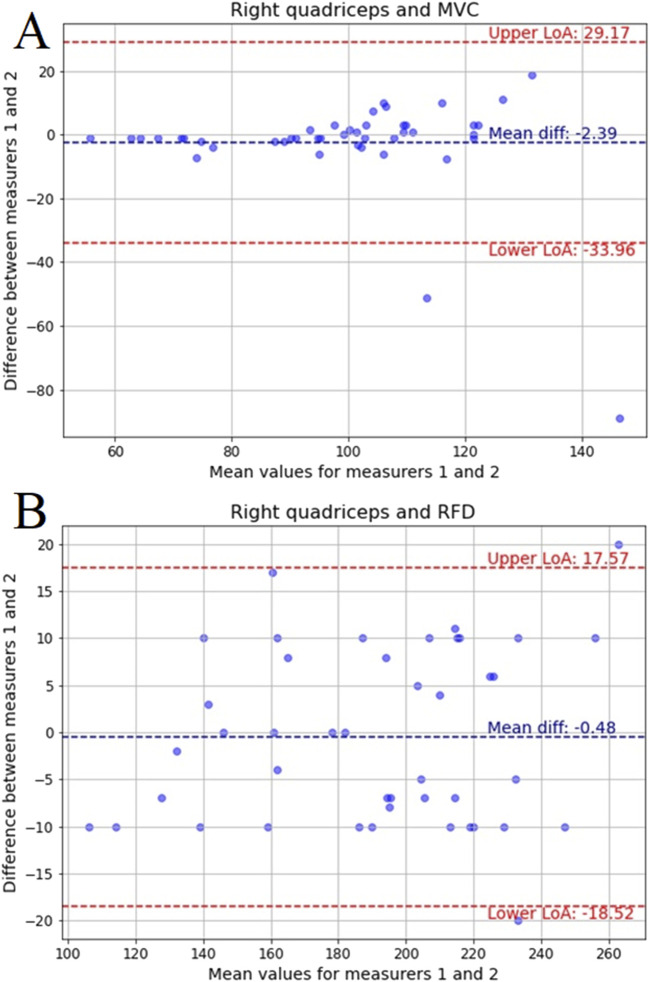
Bland-Altman plots for: **(A)** MVC of the right quadriceps and **(B)** RFD of the right quadriceps recorded by two measurers with mean difference and limits of agreement (LoA) for 95%.

Figure 9A depicts the Bland-Altman plot for MVC in the right quadriceps, where the mean difference is −2.39, and the LoA extends from −33.96 to 29.17. The broader limits compared to the left quadriceps suggest more variation between measurers, reflecting the previously observed higher MAE and MSE values for MVC in the right quadriceps. These results further support the trend of greater variability in right-side measurements found in previous statistical analyses.

Figure 9B displays the Bland-Altman plot for RFD in the right quadriceps, with a mean difference of −0.48 and LoA from −18.52 to 17.57. While the bias is negligible, the spread of differences is slightly wider than in the left quadriceps, mirroring trends from MAE and MSE values. Despite this, the overall agreement remains strong, supporting the findings from ICC and boxplots that demonstrated high reliability across all measurements.

## 4 Discussion

The findings of this study suggest the reliability and consistency of the K-push handheld dynamometer for measuring both MVC and RFD in the quadriceps. The minimal inter-examiner variability suggests that the instrument provides stable and reproducible results regardless of who performs the measurement. Additionally, the comparison of muscle strength and force development across different groups revealed that athletes, particularly footballers and MMA fighters, exhibit superior and more consistent muscle performance than the general non-training group. This highlights the importance of using a reliable measurement tool like the K-push dynamometer to accurately assess and compare muscle characteristics across diverse populations, including those with different athletic backgrounds. Ultimately, these results reinforce the K-push handheld dynamometer as a valuable tool for both clinical and sports science assessments.

The study found that while measurements of quadriceps performance, specifically MVC and RFD, were generally consistent, some variability existed between the left and right quadriceps. Our results align with previous studies demonstrating the reliable assessment of force, torque, and normalized torque using the Kinvent handheld dynamometer in athletic shoulder tests (Olds et al., 2023). The left side showed more reliable measurements with smaller errors, whereas the right side exhibited greater inconsistency, particularly in MVC. This was reflected in the higher variability in MAE and MSE for the right quadriceps. These findings suggest that the right quadriceps may be more prone to measurement fluctuations, which could impact the accuracy of performance assessments. The results highlight the need for careful consideration of side-to-side differences when evaluating muscle function, as this variability could affect the interpretation of strength and force development data, and may require refined techniques or additional focus on the right side in both clinical and research settings, namely using asymmetry indexes (Bishop et al., 2017).

When examining group comparisons, athletes from football and MMA showed highly similar MVC and RFD values, with minimal differences between the two groups. In contrast, the general non-training group exhibited more variability in both MVC and RFD for both left and right quadriceps, with significant differences when compared to athletes, particularly MMA fighters. For instance, non-athletes showed significantly higher variability in RFD compared to athletes, especially MMA fighters. These results reveal the distinct muscle characteristics associated with athletic training and highlight the importance of considering group-specific differences when assessing muscle performance, as athletes showed much less variability than the general population (Lisee et al., 2019). These findings suggest that measurements in non-athletes may require additional trials to enhance familiarization and stabilize performance due to the greater inconsistencies observed. This aligns with previous research (Rizzato et al., 2024) indicating that sports practice enhances muscle recruitment strategies, improving accuracy in isometric tasks. Furthermore, the accuracy of force production appears intensity-dependent, with lower accuracy at higher intensities.

The study showed very high reliability in the measurements of MVC and RFD for both the left and right quadriceps, with strong ICCs indicating excellent consistency between measurers. These results are interesting, as some studies suggest that repeated measurements can impact data reproducibility (Krause et al., 2014). Left quadriceps MVC showed near-perfect reliability, while RFD reliability was also very high, although with slightly more variability, particularly for the second ICC model. Right quadriceps MVC showed slightly lower reliability, though still strong, with more variability indicated by wider confidence intervals. RFD for the right quadriceps showed near-ideal consistency, with perfect agreement between measurers. This level of reliability is especially important in clinical and research settings where precise assessments of muscle strength are necessary for monitoring progress or evaluating interventions and in which previous studies suggest limitation with movements where participants can overpower the testers (Kelln et al., 2008).

This study is not without limitations. The sample population, while diverse in athletic background, may not fully represent the general population, potentially limiting the broader applicability of the findings. Additionally, the focus on only the quadriceps muscle group restricts the generalization of the conclusions regarding the reliability of the K-push dynamometer for other muscle groups. A lack of comparison with gold standard or other device can be also treated as a limitation of this study. Future research should investigate the device’s reliability and inter-limb variability in different muscle groups and across more diverse populations, including elderly individuals or those with neuromuscular disorders. Furthermore, exploring the impact of different testing protocols and rater experience levels on measurement variability would provide a more comprehensive understanding of the device’s utility.

Despite the limitations, this study revealed the reliability and consistency of the K-push handheld dynamometer suggesting its potential as a valuable tool for clinicians and practitioners in examining quadriceps strength and power. Its ease of use and portability make it particularly suitable for field-based assessments and remote monitoring, allowing for convenient and frequent tracking of muscle function. The slight inter-limb variability observed in this study highlights the need to assess each limb independently. Instead of relying solely on bilateral comparisons, clinicians should establish individual baselines for each limb and track changes over time to provide a more accurate and personalized assessment of muscle function.

## 5 Conclusion

In conclusion, this study establishes that MVC and RFD measurements in the quadriceps, obtained using the K-push handheld dynamometer, are highly reliable and consistent, with minimal inter-examiner variability. The results showed nearly perfect correlations, particularly for the left quadriceps, and very high intraclass correlation coefficients, especially for RFD, indicating strong agreement between the two measurers. Furthermore, the non-significant p-values for MVC and RFD measurements across both left and right quadriceps suggest that the instrument provides consistent and reproducible results regardless of the examiner. The Bland-Altman plots confirmed high inter-rater agreement, especially for MVC measurements, with only slight variability observed in RFD and right-side data. Combined with supporting statistical metrics, these results validate the consistency and robustness of the measurement approach. These findings confirms the reliability of the K-push handheld dynamometer as an accurate tool for assessing muscle strength and force development, even with multiple examiners.

Additionally, the study highlights the variability in muscle performance between different groups, with athletes (particularly MMA fighters and footballers) showing significantly higher strength and RFD compared to the general non-training group, while the latter exhibited greater variability in both parameters. These group differences further support the need for consistent measurement tools like the K-push dynamometer to accurately assess muscle characteristics across diverse populations. Overall, the K-push handheld dynamometer shows to be a reliable and effective device for assessing quadriceps performance, providing valuable alternatives into muscle function that can be applied in both clinical settings and sports science research.

## Data Availability

The raw data supporting the conclusions of this article will be made available by the authors, without undue reservation.
